# Syndrome d’hyperéosinophilie avec atteinte cardiaque

**DOI:** 10.11604/pamj.2017.28.270.14316

**Published:** 2017-11-28

**Authors:** Ihsen Zairi, Khadija Mzoughi

**Affiliations:** 1 Service de Cardiologie, Hôpital Habib Thameur, Tunis

**Keywords:** Hyperéosinophilie, fibrose endomyocardique, échocardiographie, Hypereosinophilia, endomyocardial fibrosis, echocardiography

## Abstract

We report the case of a 30-year old man with no previous medical history and without cardiovascular risk factors, hospitalized for NYHA Class IV dyspnoea associated with palpitations in the Department of Cardiology. Clinical examination showed signs of cardiac decompensation. The patient didn't have fever, hepatosplenomegaly and palpable lymphadenopathies. Laboratory tests showed hypereosinophilia greater than 4000 eosinophils per cubic millimeter. Electrocardiogram objectified atrial tachycardia at a rate of 100 beats per minute. Echocardiographic examination showed good left ventricular function, dilation of both atria and a thrombus filling the right ventricular apex. The diagnosis of thrombus lining the ventricular apex was suspected (A, B); successive exams showed progressive filling of the right ventricular apex. Cardiac MRI showed apical filling of both ventricles and after intravenous injection of gadolinium, late subendocardial enhancement in the left ventricular apex and in the right ventricular apex, suggesting fibrosis (C, D). Etiologic investigation of hypereosinophilia excluded parasitic diseases, drug therapies, neoplasias and autoimmune diseases as a cause. The diagnosis of idiopathic hypereosinophilic syndrome associated with cardiac involvement was retained. Corticosteroid therapy associated with hydroxyurea therapy and long-term anticoagulant therapy were started. Patient's evolution was gradually favorable with disappearance of hypereosinophilia.

## Image en médecine

Un homme âgé de 30 ans sans antécédents et sans facteurs de risque cardiovasculaires, a été hospitalisé en cardiologie pour une dyspnée classe IV de la New York Heart Association et palpitations. L'examen clinique retrouvait un patient qui présentait des signes de décompensation cardiaque. Le patient n'était pas fébrile et il n'existait pas d'hépatosplénomégalie, ni d'adénopathies palpables. Le bilan biologique révélait une hyperéosinophilie supérieure à 4000 polynucléaires éosinophiles par millimètre cube. L'électrocardiogramme a objectivé une tachycardie atriale à 100 battements par minute. L'échocardiographie montrait une bonne fonction ventriculaire gauche, une dilatation des deux oreillettes et un comblement de l'apex du ventricule droit. Le diagnostic de thrombus tapissant l'apex a été suspecté (A, B); les contrôles successifs montraient un comblement progressif de l'apex du ventricule droit. L'IRM cardiaque retrouvait un comblement apicale des deux ventricules et mettait en évidence après injection de gadolinium un rehaussement tardif sous-endocardique de l'apex du ventricule gauche et du ventricule droit traduisant la fibrose (C, D). La démarche diagnostique a été complétée par un bilan étiologique de l'hyperéosinophilie éliminait les causes parasitaires, médicamenteuses, les néoplasies et les maladies auto-immunes. Le diagnostic de syndrome d'hyperéosinophilie idiopathique associé à une atteinte cardiaque était retenu. Une corticothérapie était débutée en association avec l'hydroxyurée et un traitement anticoagulant au long cours. L'évolution était progressivement favorable avec disparition de l'hyperéosinophilie.

**Figure 1:**
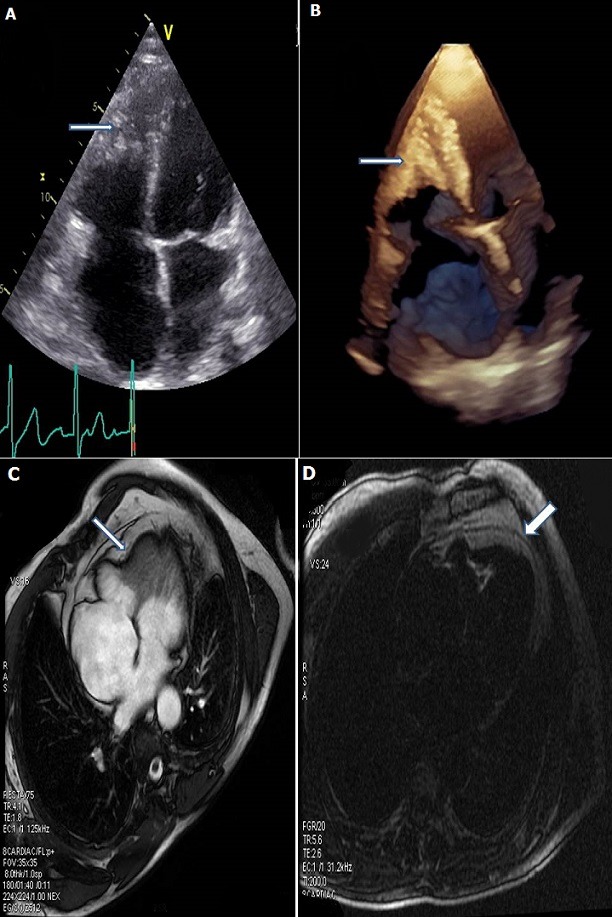
(A) échographie transthoracique coupe apicale 4 cavités (bidimensionnelle A et tridimensionnelle; (B) comblement de l'apex du ventricule droit; (C) imagerie par résonance magnétique nucléaire: un comblement apicale des deux ventricules; (D) évidence après injection de gadolinium un rehaussement tardif sous-endocardique de l'apex du ventricule gauche et du ventricule droit traduisant la fibrose

